# Supplementary material for: WordCommentsAnalyzer: A windows software tool for qualitative research
Source: F1000Res. 2018 Sep 4;7:536. Originally published 2018 May 3. [Version 2] doi: 10.12688/f1000research.14819.2 (PMC6137411; doi:10.12688/f1000research.14819.2)
Supplement: Supplementary file 3 [file f1000research-7-17614-s0002.tgz › b2a79496-4ada-47c4-9682-a99ce867836d.zip!/Eight Iranian Health Journals/Salmand_Iranian Journal of Ageing _46.docx]

© Salmand_Iranian Journal of Ageing . This work is licensed under CC BY-NC 4.0 (https://creativecommons.org/licenses/by-nc/4.0/).

Volume & Issue: دوره 1 - شماره 2

Title: اقتصاد بهداشت و سالمندی

http://salmandj.uswr.ac.ir/article-1-17-fa.html

اهداف: در این مقاله، سالمندی در ابعاد مختلف آن مورد بحث قرار می گیرد، تعریف ویژگی های زندگی سالمندی به لحاظ فردی و اجتماعی و تحولات ناظر به سالمندی در رابطه با نگرش به آن و عناصر اصلی تعیین‌کننده آن توضیح داده و تبیین می شود. بیماری و بار بیماری دوران سالمندی به عنوان یک موضوع محوری در عرصه اقتصاد سلامت و سالمندان مورد تاکید قرار می‌گیرد. همچنین عوامل موثر در رشد سالمندی و سیاست‌گذاری‌های ناظر بر آن و مسئولیت سیاستگذاران در این خصوص تحلیل می‌شود. درمجموع و در نهایت تلاش بر این خواهد بود که سالمندی، ناتوانی و هزینه‌های مراقبت‌های طولانی مدت در جدول مسائل و پدیده‌های اجتماعی ازجمله کیفیت ارتباط متقابل بین سالمندان و جامعه و نگرش به سالمندی و عواقب آن و همچنین تضاد نسل‌ها مورد توجه و بحث قرار گیرد. مواد و روش‌ها: گفتنی است سازمان جهانی بهداشت در آوریل 1995 برنامه ای را در زمینه سالمندی و سلامت راه‌اندازی کرد و در آن بیش از سالمندی بر سالم پیرشدن تاکید نمود. اجزای کلیدی برنامه شامل: فراهم‌کردن و تقویت اطلاعات پایه، نشر و تبادل اطلاعات، حمایت، طراحی و سازماندهی برنامه‌های جامعه‌نگر، پژوهش، آموزش و توسعه سیاست‌ها و خط‌مشی‌ها بود و هدف غایی تمام این برنامه‌ها پیشبرد سلامت عمومی و ارتقای کیفیت زندگی، به‌ویژه در سالمندی می‌باشد که تا حدودی چهارچوب مقاله حاضر از آن متاثر می‌باشد. یافته‌ها:‌ ما نیاز به بازبینی جامعی از نگرش موجود به سالمندی، بهداشت سالمندان و نقش آنان در خانواده و جامعه داریم. امید است که این بازبینی موفق به تغییر نگرش‌های غلط درمورد سالمندان شود. همچنین از این طریق باید سالمندان موفق به ادامه زندگی پر بار، آگاه از جایگاه و نقش با اهمیت خود در جامعه شوند. نتیجه‌گیری:‌ ارائه نقطه نظراتی از بزرگان عالم اندیشه و ادبیات مکتوب ملی و بین‌المللی البته به تبیین ابعاد سالمندی و روشن‌ترنمودن نقاط تاریک آن در این خصوص به ما کمک خواهد کرد. در مجموع با نگرش جامع به مسئله سالمندی و سالمندان، انتظار می‌رود که ارائه تصویری نسبتا جامع از وضعیت موجود، جست‌وجو برای کشف خلأهای موجود در عرصه‌های شناخت و ارایه خدمات برای بخش وسیعی از جمعیت رو به رشد سالمندی میسر و مقدور گردد.

Title: سیاست‌ های اجتماعی برای سالمندان در ژاپن و سوئد و الگوی مناسب برای سالمندان ایران

http://salmandj.uswr.ac.ir/article-1-18-fa.html

اهداف:‌ سالمندی جمعیت، هم اکنون در کشورهای توسعه یافته پدیده جدیدی نیست ولی کشورهای درحال توسعه و ازجمله ایران، از هم اکنون باید برای رفاه سالمندان آینده خود، چاره ای بیندیشند. بر اثر تحلیل رفتن قوای جسمانی و ذهنی سالخوردگان، تنوع و کثرت مسائل در آن‌ها، امری کاملا طبیعی به نظر می رسد. از این‌رو، شاید هیچ دوره ای از زندگی مانند سالخوردگی پر مساله و نگران کننده نباشد. به همین دلیل، سال‌خوردگان برای سالم زیستن، به دریافت حمایت‌های خاص که از سیاست‌های اجتماعی هدفمند سرچشمه بگیرد، نیاز دارند. در این مقاله سعی می‌شود تا با بیان نمونه‌هایی از تجربیات دو کشور ژاپن و سوئد در زمینه رفاه سالمندان، الگوی مناسبی برای سالمندان در ایران ارائه شود. مواد و روش‌ها: یکی از مشابهت‌های قابل توجه در دو کشور ژاپن و ایران، ادامه حفظ سنت‌ها در فرایند توسعه صنعتی است که در کشور ژاپن به‌خوبی انجام شد و هنوز هم این ویژگی در این کشور صنعتی توسعه‌یافته قابل مشاهده است. در ایران نیز، توسعه صنعتی هنوز در کنار فرهنگ سنتی در حال حرکت است.  یافته‌ها: سیاست‌های جدید کشور ژاپن می‌تواند در سیاست‌گذاری برای سالمندان در ایران بسترهای موجود اقتصادی و فرهنگی در جامعه، خانواده و سایر نهادهای اجتماعی را به‌کار بگیرد و برنامه‌های حمایتی فراگیر را به اکثریت سالمندان ارائه نماید. یکی از اصول مهم سیاست‌های سوئد در قبال سالمندان، تاکید بر نگهداری سالمندان در منزل تا حد ممکن است. حتی زمانی که آن‌ها نیاز به مراقبت‌های بهداشتی گسترده دارند. نتیجه‌گیری: پیشنهاد مشخص این مقاله این است که فراگیرشدن بیمه‌های اجتماعی و بازنشستگی برای تمام افراد 60 سال به بالای کشور، اعم از زنان و مردان می‌تواند یکی از بسترهای مناسب برای حمایت از سالمندان را فراهم نماید. در این سیاست، منبع عظیم خانواده و قدرت فراگیر دیگر نهادهای سنتی که عمدتا غیر دولتی هم هستند، می‌توانند امکانات بسیار وسیعی را فراهم نمایند. مهم‌ترین دلیل ارائه این سیاست‌ها در ایران، محدودیت‌های امکانات در حمایت از سالمندان است. اگر سیاست‌های مربوط به سالمندان بخواهد دنباله‌رو سیاست‌های قدیمی جهان غرب باشد، نتیجه آن ارائه برخی خدمات نمایشی به تعداد محدودی از سالمندان آن هم در شهرهاست. درحالی‌که توجه به استفاده از تجارب کشورهای توسعه‌یافته مانند سوئد و ژاپن می تواند به پژوهشگران و برنامه‌ریزان ایرانی کمک کند تا در انجام تحقیقات خودشان در زمینه سالمندان، شاخص‌های عینی و موجود در کشورهایی با نرخ سالمندی را مورد استفاده قرار دهند.

Title: ویژگی های زمین‌خوردن در سالمندان مقیم آسایشگاه

http://salmandj.uswr.ac.ir/article-1-19-fa.html

اهداف: سقوط یا زمین‌خوردن، یکی از علل شایع معلولیت و مرگ و میر در میان سالمندان، به ویژه در میان سالمندان مقیم آسایشگاه ها، محسوب می شود. جراحات بافت نرم، شکستگی‌های متعدد به ویژه شکستگی گردن استخوان ران، مچ دست، و زخم بستر از عوارض شایع آن می باشد. بررسی و توصیف ویژگی های زمین خوردن در میان سالمندان مقیم آسایگاه ها به منظور درک و شناخت علل آن و اقدام در جهت رفع آن. مواد و روش‌ها: جمعا 76 مورد سقوط به زمین در میان سالمندان مقیم آسایشگاه کهریزک تهران که از سطح عملکرد جسمانی و روانی طبیعی برخوردار بودند و در طول 6 ماه اتفاق افتاده بود (فروردین 1385 لغایت شهریور 1385) بررسی شد. برای هر مورد، تحقیق از بیمار و پرستاران و پزشک معالج و پرونده های بیماران به عمل آمد. یافته‌ها: میانگین سنی سالمندانی که دچار سقوط به زمین شده بودند 76.9 سال و کل تعداد سقوط مربوط به 29 نفر از سالمندان بوده است. این تعداد شامل 14 نفر زن معادل 48.3درصد و 15 نفر مرد سالمند معادل 51.7درصد بوده اند. 57.6درصد از موارد سقوط بدون مشکل برای سالمند بوده است و وی توانسته است پس از سقوط به تنهایی به‌پاخیزد. 42.4درصد از موارد فرد سالمند پس از سقوط قادر به برخاستن نبوده و نیاز به کمک داشته است. 2.6درصد از موارد سقوط منجر به عوارض شدید و شکستگی شده است (شکستگی جمجمه، گردن و استخوان ران). 30.3درصد محل سقوط حیاط آسایشگاه، 28.9درصد اتاق سالمند، 18.4درصد راهروها، 14.5درصد در دستشویی و توالت، 3.9درصد در حمام، 3.9درصد در سالن غذاخوری بوده است. ازنظر زمانی 45.3درصد وقوع سقوط در هنگاه صبح بوده است (7-12 قبل از ظهر) و 13.2درصد در هنگاه صرف غذا (12-14)، 17درصد در بعدازظهر (14-19)، 11.2درصد در هنگام شب (19-4 صبح) و 13.2درصد در ساعات اولیه صبح (4-7 صبح) رخ داده است. نتیجه‌گیری:‌ زمین خوردن در میان سالمندان به طور عمده، در خارج از اتاق اتفاق می افتد و در هنگامی که آن‌ها بیشتری فعالیت را دارند (مانند صبح) رخ می دهد. میزان شیوع سقوط در افرادی که سن بالاتری دارند بیشتر است. عوارض و عواقب سقوط گاهی شدید است و منجر به شکستگی جمجمه یا لگن و سر استخوان ران می‌گردد و می‌تواند باعث مرگ شود. مراقبت از عوامل خطر محیطی، می‌تواند در کاهش سقوط و عوارض معلولیت و مرگ و میر ناشی از آن در سالمندان موثر باشد.

Title: فراوانی اختلالات روانی در مراجعان سرپایی کلینیک سالمندان پویا

http://salmandj.uswr.ac.ir/article-1-20-fa.html

اهداف: یافته‌های به‌دست‌آمده از مطالعات اپیدمیولوژی غربی در زمینه شیوع اختلالات روانی در سالمندان به دلیل شرایط متفاوت اجتماعی، خصوصا عوامل فرهنگی، به کشورهای شرقی و در حال توسعه تعمیم پذیر نیست، لذا انجام مطالعات بومی اجتناب ناپذیر خواهد بود. هدف این مطالعه بررسی میزان شیوع اختلالات روانی در سالمندان است. مواد و روش‌ها: پژوهش توصیفی حاضر به منظور برآورد اولیه شیوع اختلالات روانی در سالمندان دچار مشکلات بهداشتی و نیاز آنان به خدمات روانپزشکی و به روش مقطعی انجام گردید. 100 بیمار از 314 مراجعه‌کننده پی‌در‌پی کلینیک سالمندان پویا که در ارزیابی اولیه نیازمند خدمات روانپزشکی تشخیص داده شده بودند، تحت ارزیابی دقیق بالینی و فرابالینی قرار گرفتند. یافته‌ها: یافته‌ها نشان دادند که اختلالات افسردگی (58درصد)، شناختی (29درصد) و اضطرابی (12درصد) به ترتیب فروان‌ترین اختلالات روانی بودند. در 52درصد از موارد شروع اختلال با استرس‌های زندگی مقارن بود. ابتلا به بیماری‌های جسمی همزمان شایع و پرفشاری خون (31درصد) فراوان‌ترین آن‌ها بود. مصرف داروهای متعدد پدیده‌ای تقریبا همه‌گیر بود (m=1/4±2/89). نتیجه‌گیری: یافته‌های فوق نشان‌دهنده نیاز گسترده سالمندان به خدمات بهداشتی روان در سطوح تشخیصی و درمانی به‌دلیل وجود بیماری‌های همزمان و مصرف داروهاست. با توجه به موارد فوق آموزش‌های تخصصی بیشتر در زمینه سالمندی برای پزشکان و پیراپزشکان پیشنهاد می‌شود.

Title: بررسی ارتباط بین اختلالات شناختی و افسردگی در سالمندان

http://salmandj.uswr.ac.ir/article-1-21-fa.html

اهداف: افسردگی و اختلال شناختی از مشکلات شایع سالمندان است که هر دو مشکل موجب افت کارایی، افت کیفیت زندگی و افزایش مرگ و میر می‌شود. هدف از مطالعه حاضر تعیین ارتباط بین افسردگی و سلامت شناختی در سالمندان جامعه است. مواد و روش‌ها: این مطالعه به صورت مقطعی مقایسه‌ای انجام شد. در این پژوهش از پرسش‌نامه بررسی سلامت شناختی ام‌ام‌اس‌ای و پرسش‌نامه مقیاس افسردگی سالمندان برای بررسی سلامت شناختی و افسردگی 120 سالمند شرکت‌کننده در مطالعه استفاده شد. پس از آن ضمن بررسی شیوع اختلال افسردگی و سلامت شناختی بر اساس مشخصات دموگرافیک سالمندان، با استفاده از آزمون همبستگی پیرسون، ارتباط بین افسردگی و سلامت شناختی مورد بررسی قرار گرفت. یافته‌ها: یافته‌ها نشان داد که در سطح معنی‌داری 0/01 ارتباط معنی‌داری بین افسردگی و اختلال شناختی وجود دارد و این نتایج با یافته‌های مطالعات قبلی همخوانی دارد. نتیجه‌گیری: ارتباط بین افسردگی و سلامت شناختی ریشه در نظریه تغییرات شناختی وابسته به سن قطعه پیشانی دارد. مسیر قشری، استریاتومی، پالیدمی، تالاموسی و قشری قویا با عملکردهای شناختی اجرایی قطعه پیشانی در ارتباط است. در افراد افسرده دچار ناکارآمدی می‌شود. نتیجه کاربردی که از این مطالعه و با کمک مطالعات آتی مداخله‌ای می‌توان گرفت این است که توانبخشی شناختی می‌تواند به عنوان درمانی برای افسردگی سالمندان مطرح شود.

Title: بررسی کیفیت زندگی افراد مبتلا به درد ناحیه پایین کمر

http://salmandj.uswr.ac.ir/article-1-22-fa.html

اهداف: ذکر این سوال که چرا گروهی از افراد در زندگی حتی در دوران پیری از یک سلامت نسبی برخوردار هستند و گروهی دچار معلولیت و بیماری بوده و نیازمند خدمات بهداشتی و درمانی هستند. ناتوانی ناشی از LBP تقریبا برابر با یک‌چهارم بزرگ‌سالان را سالانه تحت تاثیر قرار می دهد. شیوع کمردرد در کل جمعیت بسیار بالاست. به نحوی که گاهی تخمین زده می شود 80درصد مردم در طول زندگی به آن مبتلا می شوند. سالمندانی که مبتلا به درد ناحیه تحتانی کمر (LBP) هستند نه‌تنها از ناراحتی‌های فیزیکی رنج می‌برند، بلکه محدودیت‌های عملکردی ممکن است باعث ناتوانی آن‌ها شده و بر کیفیت زندگی آن‌ها تاثیر منفی بر جای بگذارد. این مسئله به علت افزایش موارد بیماری و هزینه‌های درمانی صرف‌شده در سال‌های اخیر بیش از پیش مورد تاکید قرار گرفته است. این پژوهش با هدف بررسی کیفیت زندگی افراد مسن و مقایسه آن با سالمندان سالم در گروه شاهد در یکی از کلینیک‌های شهر قزوین انجام شده است. مواد و روش‌ها: پژوهش حاضر یک بررسی نیمه‌تجربی با دو گروه مورد و شاهد است. تعداد افراد نمونه 56 نفر در گروه مورد است. ابزار جمع آوری اطلاعات پرسش‌نامه اطلاعات دموگرافیکی و پرسش‌نامه استاندارد کیفیت زندگی Nottingham Health profile است. درنهایت داده‌های پژوهش بانرم‌افزار SPSS و آزمون‌های آماری t، همبستگی و کای دو مورد تجزیه‌وتحلیل قرار گرفت. یافته‌ها: میانگین سنی در این بررسی 63 سال بود. کیفیت زندگی سالمندان که با پرسش‌نامه کیفیت زندگی ناتینگهام محاسبه شده است تفاوت معنی‌داری را در ابعاد انرژی (P=0/004)، خواب (P=0/018)، درد (P=0/059)، فعالیت جسمانی (P<0/001) و نمره کل کیفیت زندگی (P=0/042) در گروه مورد (سالمندان مبتلا به درد ناحیه تحتانی کمر) و گروه شاهد نشان داده است. اما در بعد انزوای اجتماعی (P=0/486) و واکنش‌های هیجانی (P=0/206) اختلاف معنی‌داری مشاهده نشد. نتیجه‌گیری: یافته‌های تحقیق نشان‌دهنده پایین‌بودن کیفیت زندگی سالمندان گروه مورد یعنی سالمندان مبتلا به درد ناحیه تحتانی کمر نسبت به گروه شاهد یعنی افراد سالم و معمولی است.

Title: بررسی اختلالات جسمی‌حرکتی سالمندان مراجعه‌کننده به مراکز توانبخشی سازمان بهزیستی شهر تهران در سال 1385

http://salmandj.uswr.ac.ir/article-1-23-fa.html

اهداف: با افزایش جمعیت سالمندان، شیوع ناتوانایی های جسمی در حال افزایش است. شیوع درد عضلانی اسکلتی در سالمندان جامعه 10 تا 71درصد تخمین زده شده است. که یک علت مهم ناتوانی زنان سالمند جامعه محسوب می‌شود. لذا هدف از انجام این تحقیق بررسی شیوع اختلالات جسمی حرکتی افراد سالمند بود. مواد و روش‌ها: پرونده‌های بایگانی‌شده سالمندان مرد و زن 60 سال به بالا که از اردیبهشت 1384 تا اردیبهشت 1385 به دو مرکز توانبخشی سازمان بهزیستی و دو بیمارستان عمومی مراجعه کرده بودند توسط یک فیزیوتراپیست مورد بررسی قرار گرفت. یافته‌ها: از 426 پرونده مورد بررسی شایع‌ترین علت مراجعه به مراکز فیزیوتراپی به‌ترتیب آرتروز زانو و کمردرد بود. همچنین درگیری زنان تقریبا سه برابر مردان بود. با رشد جمعیت سالمند زانو درد به طور اجتناب ناپذیری در حال افزایش است. این افزایش می‌تواند حاصل عوامل زیادی مثل بالارفتن سن جوامع، افزایش صدمات و رشد سریع چاقی باشد. همچنین تغییرات دیسک بین‌مهره‌ای، کاهش قدرت عضلانی پشتی و کاهش دانسیته مواد معدنی استخوان که در روند سالمندی مشاهده می‌گردد می تواند ازجمله علل موثر در کمردرد مزمن باشد. نتیجه‌گیری: زانودرد و کمردرد به ترتیب شایع‌ترین علل اختلالات و ناتوانایی‌های جسمی‌حرکتی سالمندان است.

Title: بررسی وضعیت اجتماعی و سلامت سالمندان روستایی با رویکرد طرح توانمندسازی روستایی

http://salmandj.uswr.ac.ir/article-1-24-fa.html

اهداف: این پژوهش در چهارچوب اهداف طرح توانمندسازی سالمندان روستایی و به‌منظور تعیین وضعیت موجود سلامت سالمندان روستایی (ازجمله میزان شیوع معلولیت و انواع آن و میزان شیوع بیماری‌ها و انواع آن در بین سالمندان روستایی) به قصد ارائه خدمات مختلف پیشگیرانه، پزشکی و توانبخشی به آن‌ها اجرا شده است. تعیین وضعیت اجتماعی سالمندان از ابعاد مختلف و همچنین تعیین نیازمندی‌های اساسی سالمندان روستایی برای ارائه خدمات مرتبط با آن‌ها از اهداف دیگر این پژوهش به‌شمار می‌روند. مواد و روش‌ها: در این پژوهش از روش پژوهشی توصیفی از نوع «مطالعات پیمایشی مقطعی» استفاده شده است. برای جمع‌آوری اطلاعات و داده‌ها از دو نوع پرسش‌نامه استفاده شده است: «پرسش‌نامه ارزیابی وضعیت اجتماعی و سلامت سالمندان» و «پرسش‌نامه نیازسنجی سالمندان». این طرح در بخش کورائیم شهرستان نیر از استان اردبیل اجرا شده است. این بخش 34 روستا و 14383 نفر جمعیت و 491 نفر جمعیت بالای 60 سال دارد. برای تجزیه‌وتحلیل داده‌ها از روش‌های آمار توصیفی (فراوانی و درصد) با استفاده از نرم افزار SPSS اقدام شده است. نتیجه‌گیری: نسبت سالمندی در شهرستان مذکور 3.41درصد برآورد شد. 28.8درصد از سالمندان همسران خود را از دست داده‌اند و 70.1درصد آن‌ها متأهل هستند. 14.3درصد از آن‌ها توانایی انجام امور شخصی خود را ندارند. 8.6درصد از آن‌ها به تنهایی زندگی می‌کنند. 8.4درصد از آن‌ها تحت پوشش نهادهای حمایتی هستند. 8.4درصد از آن‌ها دچار انواع مختلف معلولیت هستند، بیشترین میزان شیوع معلولیت مربوط به معلولیت شنوایی با 3.7درصد است. 50.5درصد از آن‌ها دارای انواع مختلف بیماری هستند. بیماری‌های قلبی و فشار خون با شیوع 25.3درصد شایع‌ترین نوع بیماری در بین سالمندان است. 20درصد از سالمندان، نیازمند تغذیه، پوشاک و مسکن، 19درصد نیازمند خدمات پزشکی و بهداشتی، 10درصد نیازمند خدمات توانبخشی، 41درصد نیازمند برنامه های اوقات فراغت و 10درصد نیازمند تکریم و ارزش گذاری اجتماعی هستند.

Title: تشخیص و درمان صرع در سالمندان

http://salmandj.uswr.ac.ir/article-1-25-fa.html

اهداف: تشخیص و درمان بیماری‌ها در سنین بالا نیازمند به‌کارگیری توانمندی‌های خاص است. صرع نیز از این قاعده مستثنی نیست. طبق تحقیقات انجام شده میزان بروز تشنج در افراد مسن در حال افزایش است. از طرف دیگر افزایش سنی در جامعه انسانی، باعث افزایش تعداد افراد مسن در جوامع شده است. کشور ما نیز در سال‌های نه چندان دور با این مسئله یعنی افزایش تعداد افراد مسن روبه‌روست و اگر به این گروه توجه نشود می‌تواند در آینده مشکلاتی ایجاد نماید. مواد و روش‌ها: بروز صرع را در افراد با سن 80 سال 140/100000 ذکر کرده‌اند. آنچه اهمیت دارد موضوع تشخیص بیماری در افراد مسن است؛ زیرا می‌تواند حالاتی مثل T.I.A، مشکلات قلبی و عروقی، اختلال در درک زمان، مکان و یا افراد به صورت گذرا در بیماران با تشخیص دمانس، TGA و سنکوپ عصبی .. را تقلید نماید. یافته‌ها: گاهی تابلو بروز علایم در افراد مسن با افراد جوان متفاوت است و گاهی حتی به صورت NCSE ظاهر می‌شود. از طرف دیگر تغییرات وابسته به سن که باعث بروز صرع در افراد مسن می‌شود به‌خوبی مطالعه نشده‌اند. درمان و انتخاب نوع دارو در افراد مسن توجهات خاص خود را می‌طلبد. تغییراتی مثل تغییرات متابولیک، تغییر در تحمل مغز نسبت به داروها و عوارض آن‌ها، تعییر در عملکرد ارگان‌های بدن، همزمان‌شدن بیماری‌های دیگر و مصرف داروهای متعدد برای درمان آن‌ها می‌تواند درمان ما را با چالش مواجه کند. نتیجه‌گیری: اکثر جوامع بین المللی به مسئله سالمندی اهمیت داده می‌شود و این باعث شده ما امیدوار باشیم که در آینده عوامل موثر در صرع سالمندان ازجمله تغییرات وابسته به سن که مغز را حساس به اپی لپسی می‌کند را بهتر شناخته و متعاقب آن درمان بهتری را انجام دهیم.
